# Stepwise metabolic engineering of *Candida tropicalis* for efficient xylitol production from xylose mother liquor

**DOI:** 10.1186/s12934-021-01596-1

**Published:** 2021-05-25

**Authors:** Lihua Zhang, Zhen Chen, Junhua Wang, Wei Shen, Qi Li, Xianzhong Chen

**Affiliations:** Key Laboratory of Industrial Biotechnology, Ministry of Education, School of Biotechnology, Jiangnan University, 1800 Lihu Road, Wuxi, 214122 People’s Republic of China

**Keywords:** Xylose mother liquor, *Candida tropicalis*, Cofactor regeneration, Xylitol, Fermentation optimization

## Abstract

**Background:**

Commercial xylose purification produces xylose mother liquor (XML) as a major byproduct, which has become an inexpensive and abundant carbon source. A portion of this XML has been used to produce low-value-added products such as caramel but the remainder often ends up as an organic pollutant. This has become an issue of industrial concern. In this study, a uracil-deficient *Candida tropicalis* strain was engineered to efficiently convert XML to the commercially useful product xylitol.

**Results:**

The xylitol dehydrogenase gene was deleted to block the conversion of xylitol to xylulose. Then, an NADPH regeneration system was added through heterologous expression of the *Yarrowia lipolytica* genes encoding 6-phosphate-gluconic acid dehydrogenase and 6-phosphate-glucose dehydrogenase. After process optimization, the engineered strain, *C. tropicalis* XZX-B4ZG, produced 97.10 g L^− 1^ xylitol in 120 h from 300 g L^− 1^ XML in a 5-L fermenter. The xylitol production rate was 0.82 g L^− 1 ^h^− 1^ and the conversion rate was 92.40 %.

**Conclusions:**

In conclusion, this study performed a combination of metabolic engineering and process optimizing in *C. tropicalis* to enhance xylitol production from XML. The use of *C. tropicalis* XZX-B4ZG, therefore, provided a convenient method to transform the industrial by-product XML into the useful material xylitol.

**Supplementary Information:**

The online version contains supplementary material available at 10.1186/s12934-021-01596-1.

## Background

Xylitol is a five-carbon sugar alcohol widely and increasingly used in food, medicines and the chemical industry. It is currently produced on an industrial scale via the catalytic hydrogenation of d-xylose [1]. The xylose used in this process is generally extracted from the acid hydrolysate of hemicellulose obtained from sources such as corncob and sugarcane bagasse [2]. This acid hydrolysate contains many other sugars and impurities that complicate xylose purification and lead to the production of xylose mother liquor (XML) as the major by-product of xylose manufacture [3, 4]. XML is a dark brown, nutrient-dense liquor that contains xylose (35–40%), glucose (8–10%), l-arabinose (10–15%), d-galactose (8–10%), and trace amount of other sugars [1, 5, 6]. The production of one ton of xylose results in the formation of one to one and a half tons of XML. It has been estimated that several large factories in China produce 50,000–80,000 tons of XML per year [1]. A portion of this XML has been used to produce low-value-added products such as caramel but the remainder often ends up as an organic pollutant. This has become an issue of industrial concern [7].

A common strategy to avoid pollution and improve the economic value of XML is to isolate the individual sugars using simulated moving-bed chromatography. However, the current techniques for sugar separation are inefficient, and the operating costs are high compared with the value of the sugars [1, 8]. An alternate strategy is to increase the proportion of the sugar of interest by removing other sugars through microbial fermentation. For example, Cheng et al. isolated a yeast strain that could metabolize most of the sugars, but not l-arabinose [8]. Use of this strain increased the l-arabinose content of the XML to 86.1% of total sugars, allowing the l-arabinose to be isolated using a simple process. Other microbial processes have been used to convert XML to high-value chemicals such as xylitol [1, 6], solvents [3, 9], welan gum [10] and succinic acid [7]. However, XML also contains furfural and 5-hydroxymethyl furfural, which are potent inhibitors of microbial growth. Furfurals cause the accumulation of reactive oxygen species and cellular damage in *Saccharomyces cerevisiae* [11] and increase the toxicity of phenols in *Escherichia coli* [12]. Furfurals also inhibit xylitol production from non-detoxified corncob hemicellulose acid hydrolysate [13]. Therefore, XML must be detoxified before it can be used as a fermentation feedstock. Unfortunately, the processes used to detoxify XML strip it of nutrients and increase production costs. In our opinion, there is an urgent need for simpler biotechnological systems that can improve the value of XML.

**Fig. 1:**
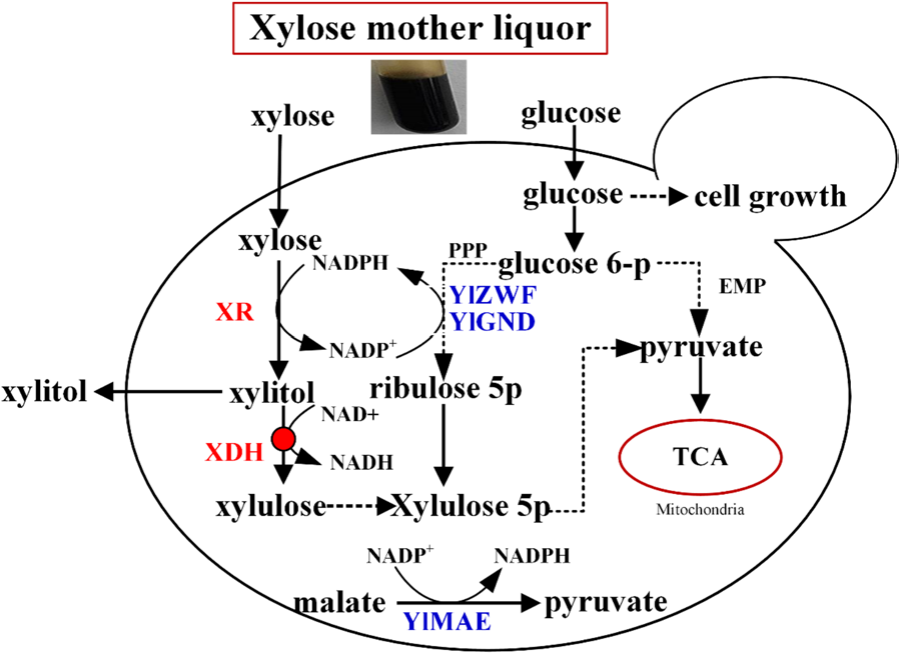
Metabolic engineering of *C. tropicalis* XZX to enhance xylitol production from XML. Deletion of *XDH* inhibits conversion of xylitol to xylulose; coenzyme regeneration is enhanced by overexpression of *YlZWF* and *YlGND* or of *YlMAE*

One potential solution that addresses the problem of XML accumulation through microbial fermentation while mitigating the need to detoxify the XML is the use of *Candida tropicalis*, a diploid yeast, to convert XML to xylitol [14]. This conversion is catalyzed by the enzyme xylose reductase, which requires NADPH as a coenzyme. Unfortunately, *C. tropicalis* also expresses the enzyme xylitol dehydrogenase (XDH), which prevents xylitol accumulation by oxidizing it to xylulose (Fig. 1). Disruption of the *XYL2* genes, which encode XDH, has been shown to increase the xylitol yield to 98 %, with a volumetric productivity of 3.23 g^− 1^ L^− 1^ h^− 1^, when pure xylose was used as the substrate [15]. The availability of NADPH is also a major limitation, as volumetric xylitol productivity was higher when NADPH regeneration was enhanced in a defined medium [16, 17]. XML was recently used as a feedstock for xylitol production by a combination of two complementary strains: wild-type *C. tropicalis* and *xylA*-disrupted *Bacillus subtilis* [1]. A conversion rate of 0.75 g g^− 1^ was obtained. In that one-pot system, *C. tropicalis* was used both to deplete furfural and 5-hydroxymethyl furfural and to convert xylose into xylitol. Research focusing on the mechanisms of furfural tolerance has shown that NADH formation during xylose metabolism (Fig. 1) facilitates the detoxification of furfural, and that the half maximal inhibitory concentration of furfural for *C. tropicalis* was much higher (3.69 g L^− 1^) than that of other microbes [18]. This enhanced furfural tolerance enhances the ability of *C. tropicalis* to process XML and other biomass hydrolysates.

In a previous study, we developed an efficient strategy for the genetic manipulation of *C. tropicalis* through sequential gene disruption and expression [19]. In this study, the xylose metabolism network of *C. tropicalis* was rationally modified to facilitate xylitol production using XML as the fermentation feedstock. Firstly, the *XYL2* genes of *C. tropicalis* strain XZX were disrupted to block the conversion of xylitol to xylulose. Secondly, two parallel NADPH coenzyme regeneration systems were constructed, one involving the pentose phosphate pathway and the other involving malate formation. Subsequent testing and process optimization yielded a simple and efficient process to produce xylitol from XML with high productivity.

## Results

### Analysis of the XML used in this study

The chemical composition of XML varies widely depending on the corn cob hydrolysis and crystallization processes used during xylose production [1]. Therefore, the composition of the XML used in this study was characterized. After being sterilized in an autoclave, the XML was found to contain 98.36 g L^− 1^ glucose, 512.31 g L^− 1^ xylose and 96.28 g L^− 1^ arabinose. Furfural, acetate and phenol were also detected. The acetate and phenol concentrations were very low, but the furan compounds (furfural and 5-hydroxymethyl furfural) concentration in XML was 7.3 g L^− 1^ (of which furfural was 4.2 g L^− 1^). The effect of compounds such as furfural and acetate on the growth of *C. tropicalis* cells was investigated using 200 g L^− 1^ (w/v) XML as the carbon source (furfural 0.82 g L^− 1^, 5-hydroxymethyl furfural 0.65 g L^− 1^). Although reports have stated that furfural can seriously suppress the growth of many microorganisms [13, 18], we found that *C. tropicalis* XZX could grow at a rapid rate (see Additional file 1: Fig. S1), consistent with previous studies showing that *C. tropicalis* is tolerance to these compounds [18]. These results suggested that *C. tropicalis* XZX could be transformed into a useful host for xylitol production from XML.

### **Characterization of*XYL2*-disrupted strain**

For *C. tropicalis* XZX to accumulate useful quantities of xylitol, xylitol dehydrogenase activity must be eliminated through gene deletion (Fig. 1). The two *XYL2* alleles were sequentially deleted using the established genetic manipulation strategy [19]. A series of mutant strains were constructed during this process, including *C. tropicalis* XZX-B1 (*ura3*/*ura3 xyl2::gda-URA3*/*XYL2*), *C. tropicalis* XZX-B2 (*ura3*/*ura3 xyl2*::*gda*/*XYL2*), *C. tropicalis* XZX-B3 (*ura3*/*ura3 xyl2*::*gda*/*xyl2*::*gda*-*URA3*) and *C. tropicalis* XZX-B4 (*ura3*/*ura3 xyl2*::*gda*/*xyl2*::*gda*).

**Fig. 2:**
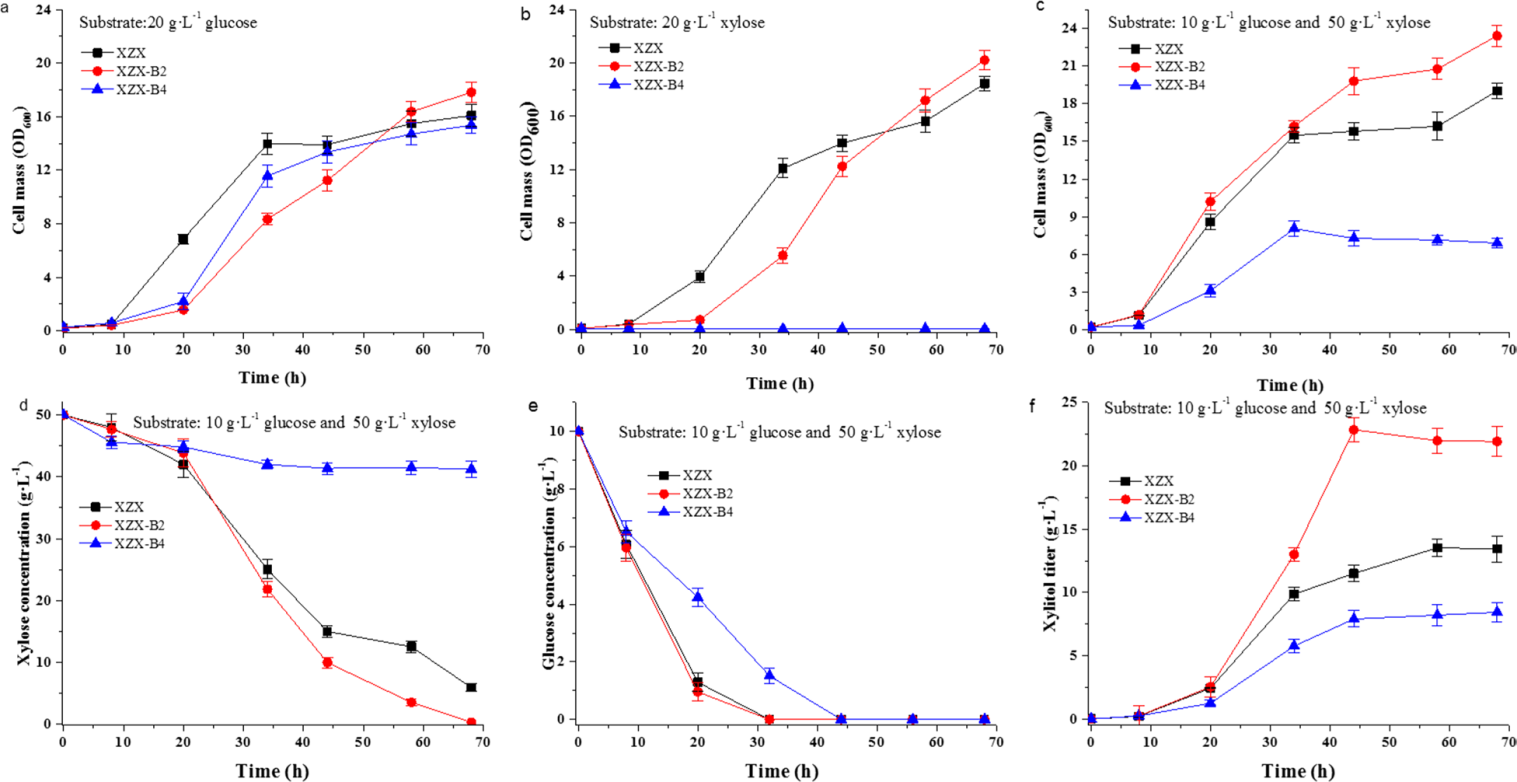
Characterization of strains XZX-B2, XZX-B4 and control strain XZX. Growth patterns were evaluated using: **a** glucose, **b** xylose, or **c** a mixture of glucose and xylose as the carbon source. Evaluation of (**d**) xylose consumption, (**e**) glucose consumption, and (**f**) xylitol titer when using a mixture of glucose and xylose as the carbon source. Error bars indicate standard deviations from the mean of triplicate biological replicates

*C. tropicalis* strains XZX, XZX-B2 and XZX-B4 were cultured at 30 °C for 3 days on SM plates containing xylose as the sole carbon source. Strains XZX (control) and XZX-B2 grew well, but XZX-B4 was unable to grow (see Additional file 1: Fig. S2). The control and XZX-B2 exhibited similar growth rates and final cell concentrations in liquid SM containing xylose as the sole carbon source. However, the growth of XZX-B4 was completely inhibited by the deletion of both *XYL2* allelic genes (Fig. 2b). In contrast, deletion of both *XYL2* alleles had only a slight impact on growth when glucose was used as the sole carbon source (Fig. 2a). Thus, deleting one *XYL2* gene had no effect on xylose utilization and cell growth as the other *XYL2* gene could complement xylose metabolism. However, deletion of both *XYL2* genes eliminated the cell’s ability to grow when using xylose as the sole carbon source.

In our previous study, the xylitol accumulation of *XYL2*-disrupted strains was evaluated using a mixture of glucose and xylose as the carbon source (Fig. 2c, d, f) [20]. Strains XZX and XZX-B2 grew at a rate higher than XZX-B4 (Fig. 2c). They continued to grow, using xylose as the carbon source, when glucose was depleted (Fig. 2d, e). In contrast, XZX-B4 stopped growing after glucose was depleted and yielded a lower final cell mass (Fig. 2c, d). Furthermore, the xylitol titer of XZX-B2 was higher than those of XZX and XZX-B4 (Fig. 2f). Interestingly, the conversion rate (xylitol titer versus xylose consumed) of XZX-B4 reached 99 %, which is near the theoretical value (100 %) and much higher than those of the other two strains (Fig. 2d, f).

### **Increasing coenzyme regeneration in*C. tropicalis***

Since xylose reductase uses NADPH preferentially or exclusively as a coenzyme [21, 22] (Fig. 1), the low xylitol titer observed with XZX-B4 may have been caused by a low intracellular NADPH concentration. To test this hypothesis, two different coenzyme regeneration systems were created and their impact on xylitol production and cell growth were investigated.

Since the pentose phosphate pathway is the primary means of NADPH generation, diversion of glycolytic flux from the Embden–Meyerhof–Parnas pathway to the pentose phosphate pathway should increase NADPH availability [23]. Therefore, two critical enzymes of the pentose phosphate pathway (glucose 6-phosphate dehydrogenase and 6-phosphogluconate dehydrogenase) were overexpressed (Fig. 1). To accomplish this, *YlZWF* and *YlGND* from the non-conventional yeast *Yarrowia lipolytica* were incorporated into separate expression cassettes that were inserted into XZX-B4 in a two-step procedure. First, the cassette containing *YlZWF* was used to transform XZX-B4, creating *C. tropicalis* XZX-B4Z. PCR (see Additional file 1: Fig. S3b) and DNA sequencing were used to confirm the genomic modification. An assessment of gene expression using qPCR indicated that *YlZWF* was being expressed (see Additional file 1: Fig. S4a). In the second step, the cassette containing *YlGND* was used to transform XZX-B4Zt, creating *C. tropicalis* XZX-B4ZG. This genomic modification was also confirmed using PCR (see Additional file 1: Fig. S3c), and *YlGND* expression was confirmed using qPCR (see Additional file 1: Fig. S4b). Meanwhile, the *YlGND* expression cassette was transformed into XZX-B, creating *C. tropicalis* XZX-B4G.

In the second NADPH regeneration system, malic enzyme was overexpressed. Malic enzyme converts the tricarboxylic acid cycle intermediate malate into pyruvate while simultaneously converting NADP^+^ to NADPH [24]. To accomplish this, *YlMAE* from *Y. lipolytica* CICC 31,251 was inserted into an expression cassette that was used to transform XZX-B4, creating *C. tropicalis* XZX-B4M. The genomic modification was confirmed by PCR (see Additional file 1: Fig. S3d) and DNA sequencing, and *YlMAE* expression was confirmed using qPCR (see Additional file 1: Fig. S4c).

**Fig. 3:**
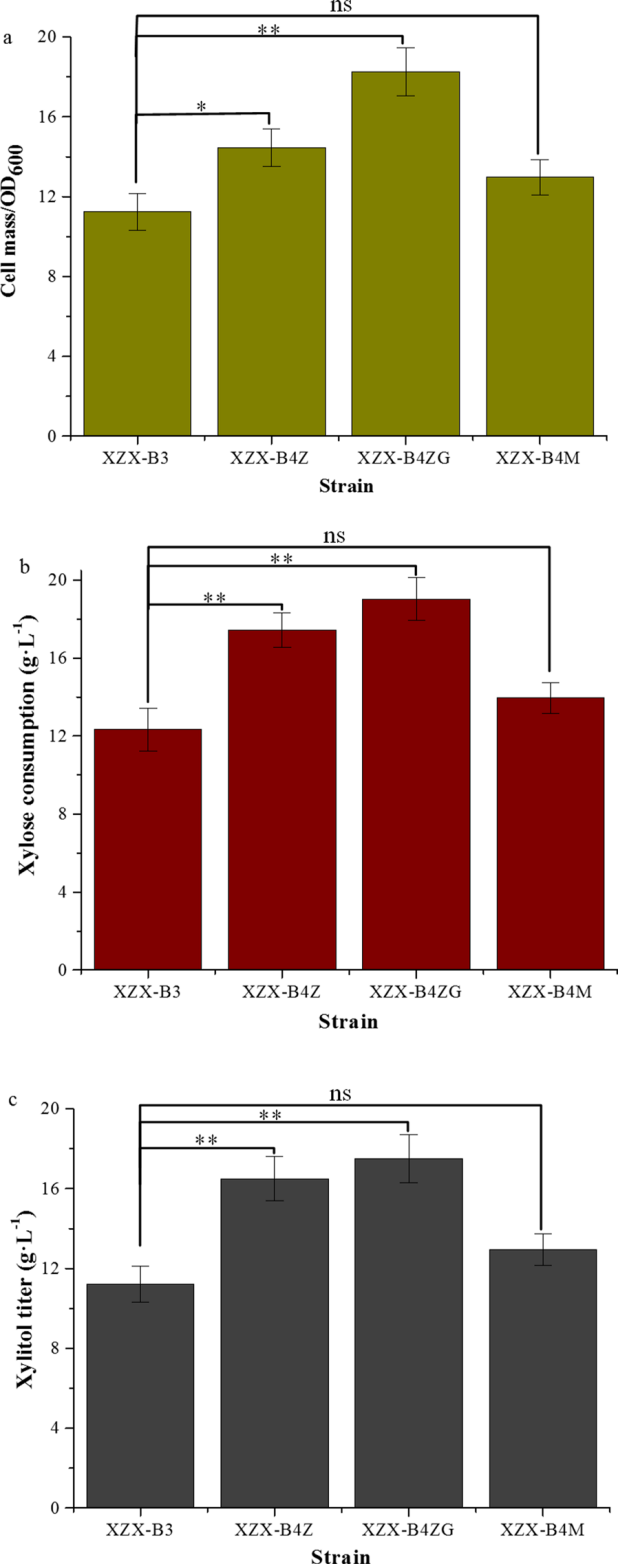
Effect of coenzyme regeneration systems. Comparisons of: **a** cell mass, **b** xylose consumption, and (**c**) xylitol titer among *C. tropicalis* strains XZX-B4Z, which expresses *YlZWF*; ZX-B4ZG, which overexpresses both *YlZWF* and *YlGND*; and XZX-B4M, which overexpresses *YlMAE*. The control strain, XZX-B3 is the precursor of parent XZX-B4 that retains the *URA3* marker. Error bars indicate standard deviations from the means of triplicate biological replicates. Differences were analyzed by Student’s *t*-test. **p < 0.01, *p < 0.05; “ns” means not significant

Strains XZX-B3, XZX-B4Z, XZX-B4ZG and XZX-B4M to assess the impact of the NADPH regeneration systems on cell growth and xylitol production. Strain XZX-B3 was used as the control, rather than strain XZX-B4, because strain XZX-B3 exhibits the *ura3*^+^ phenotype (Table 1) and displays better growth performance than XZX-B4, which is *ura3*^−^. The four strains were grown in fermentation medium (a mixture of 10 g L^− 1^ glucose and 50 g L^− 1^ xylose as the carbon source) and cell growth, xylose consumption, xylitol production, and intracellular NADPH concentration were evaluated. Strain XZX-B4ZG produced the highest cell mass and consumed the most xylose (Fig. 3a, b), producing xylitol with a titer of 17.5 g L^− 1^ with a bioconversion rate of 95.1 % (Fig. 3c). Interestingly, XZX-B4Z produced higher cell mass, xylose consumption and xylitol titer than XZX-B3 (Fig. 3). And the lag phase of XZX-B4Z and XZX-B4ZG was significantly reduced when growing in fermentation medium with 2 g L^− 1^ of furfural (see Additional file 1: Fig. S5). Previous studies demonstrated that overexpression of *ZWF1* (which encodes glucose 6-phosphate dehydrogenase) in *S. cerevisiae* allowed growth at furfural concentrations that are normally toxic, whereas a *zwf1*^−^ mutant displayed higher furfural sensitivity [25]. Our results led to a similar conclusion. Overexpression of *YlZWF* in *C. tropicalis* could play a similar role while improving xylitol production.

**Table 1:** Strains and plasmids used in this study

	Description	Reference or source^a^
Strains		
* E*. *coli* JM109	*rec*A1 *end*A1 *gyr*A96 *thi-1 hsd*Rl7 *(rk*^*−*^ *mk*^*+*^*) e14*^*−*^ *(mcr*A^−^*) sup*E44 *rel*A1 △(*lac*-*pro*AB)/F’ [*tra*D36 *pro*AB^+^ *lac*lq *lac*Z △M15]	TaKaRa
* C. tropicalis* ATCC 20,336	*URA3*/*URA3 XYL2*/*XYL2*	ATCC
* Y. lipolytica* CICC 31,251		CICC
* C. tropicalis* XZX	*ura3*/*ura3 XYL2*/*XYL2*	[19]
* C. tropicalis* XZX-B1	*ura3*/*ura3 xyl2::gda*-*URA3*/*XYL2*	This study
* C. tropicalis* XZX-B2	*ura3*/*ura3 xyl2::gda*/*XYL2*	This study
* C. tropicalis* XZX-B3	*ura3*/*ura3 xyl2::gda*/*xyl2::gda*-*URA3*	This study
* C. tropicalis* XZX-B4	*ura3*/*ura3 xyl2::gda*/*xyl2::gda*	This study
* C. tropicalis* XZX-B4G	*ura3*/*ura3 xyl2::gda*-*URA3*-*P*_*GAPDH*_-*GND*-*T*_*GAPDH*_/ *xyl2::gda*	This study
* C. tropicalis* XZX-B4Z	*ura3*/*ura3 xyl2::gda*-*URA3*-*P*_*GAPDH*_-*ZWF*-*T*_*GAPDH*_/ *xyl2::gda*	This study
* C. tropicalis* XZX-B4Zt	*ura3*/*ura3 xyl2::gda*-*P*_*GAPDH*_-*ZWF*-*T*_*GAPDH*_/*xyl2::gda*	This study
* C. tropicalis* XZX-B4ZG	*ura3*/*ura3 xyl2::gda*-*P*_*GAPDH*_-*ZWF*-*T*_*GAPDH*_/*xyl2::gda*-*URA3*-*P*_*GAPDH*_-*GND*-*T*_*GAPDH*_	This study
* C. tropicalis* XZX-B4M	*ura3*/*ura3 xyl2::gda*-*URA3-P*_*GAPDH*_-*MAE*-*T*_*GAPDH*_ /*xyl2::gda*	This study
Plasmids		
* Tm-gda-URA3*	Contain a functional marker gene flanked by *gda*	[19]
Ts*-XYL2I*-*gda-URA3*	Contain the cassette for the disruption of the first *XYL2* allele	This study
Ts*-XYL2II*-*gda-URA3*	Contain the cassette for the disruption of the second *XYL2* allele	This study
* Ts-XYL2I-gda-URA3-P*_*GAPDH*_*-YlZWF-T*_*GAPDH*_.	Contain the *YlZWF* gene expression cassette	This study
* Ts-XYL2II-gda-URA3-P*_*GAPDH*_ *-YlGND-T*_*GAPDH*_	Contain the *YlGND* gene expression cassette	This study
* Ts-XYL2I-gda-URA3-P*_*GAPDH*_ *-YlMAE-T*_*GAPDH*_	Contain the *YlMAE* gene expression cassette	This study

^a^ATCC: American Type Culture Collection; CICC: China Center of Industrial Culture Collection

### Effect of fermentation conditions on xylitol production

In initial experiments using 100 g L^− 1^ XML as the substrate, strain XZX-B4ZG produced the highest xylitol titer (25.9 ± 1.34 g L^− 1^), with a yield of 87.8 % (mol/mol; see Additional file 1: Table S1). Thus, introduction of this NADPH regeneration system had a significant impact on xylitol production from XML, supporting our hypothesis that supplying sufficient coenzyme could improve xylitol production. Despite this achievement, this xylitol titer was still lower than that desired for industrial-scale xylitol production. To meet this goal, xylitol production by XZX-B4ZG was optimized.

**Fig. 4:**
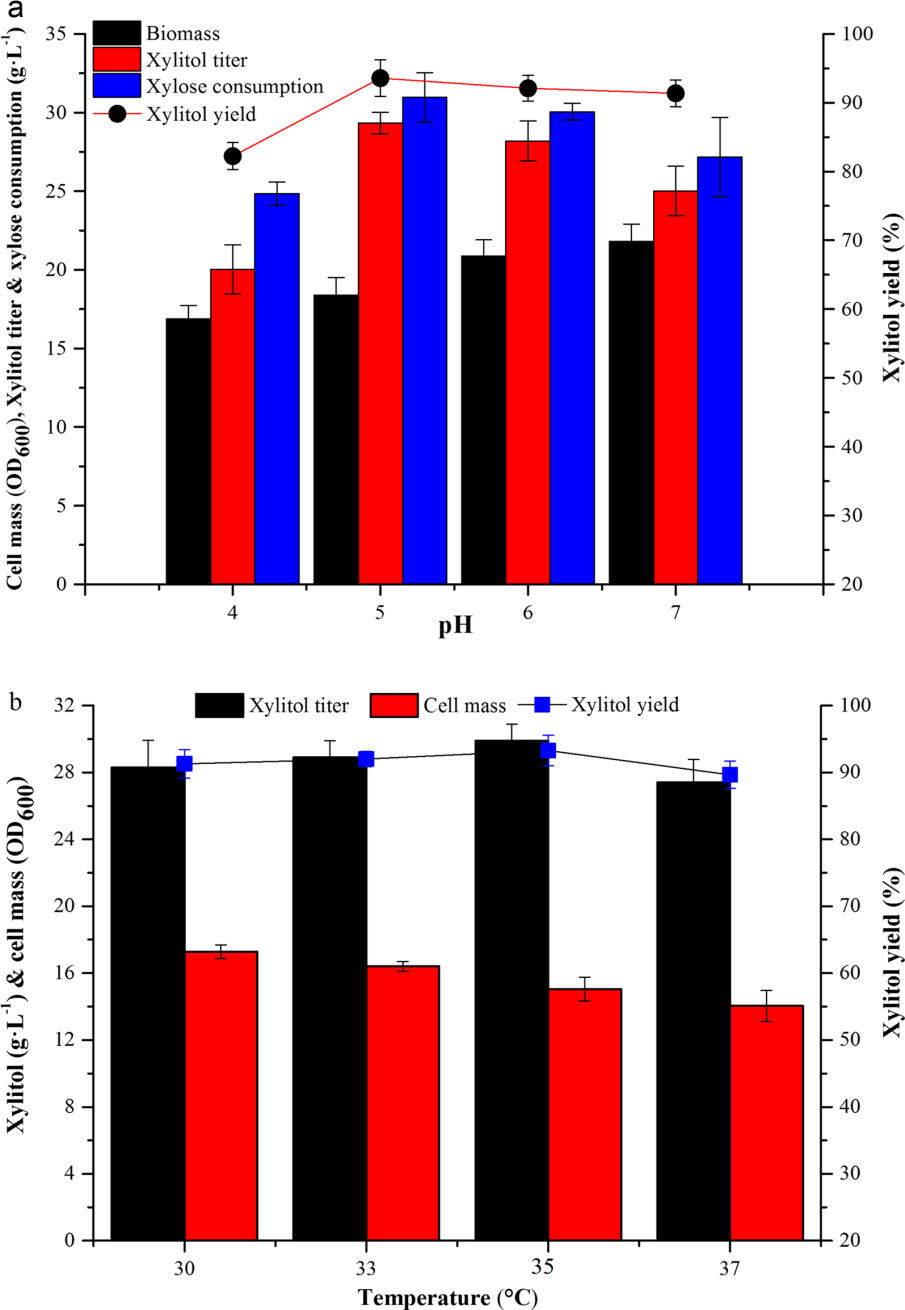
Optimization of fermentation conditions for xylitol production from XML by *C. tropicalis* XZX-B4ZG. **a** Effect of pH on biomass, xylose consumption, xylitol titer and xylitol yield. **b** Effect of temperature on biomass, xylitol titer and xylitol yield. Fermentations were conducted in shake flasks containing 100 g L^− 1^ XML. Error bars indicate standard deviations from the means of triplicate biological replicates

The effect of initial pH was evaluated using pH values from 4 to 7 (Fig. 4a). Cell mass increased with pH value over this range, but the xylitol titer (29.3 g L^− 1^) and conversion rate (93.5 %) peaked at pH 5.0, so pH 5.0 was used in subsequent fermentations. A previous study showed that the optimal temperatures for cell growth and desired product synthesis were different [26]. In this study, the effect of temperature on xylitol production by XZX-B4ZG was evaluated at 30, 33, 35 and 37 °C (Fig. 4b). Consistent with the previous study, cell mass tended to decrease with increasing temperature, but xylitol titer and yield both peaked at 35 °C. At the optimal temperature, the xylitol titer was 29.9 g L^− 1^ xylitol with a yield of 93.3 %. Therefore, subsequent fermentations were performed at 35 °C.

**Fig. 5:**
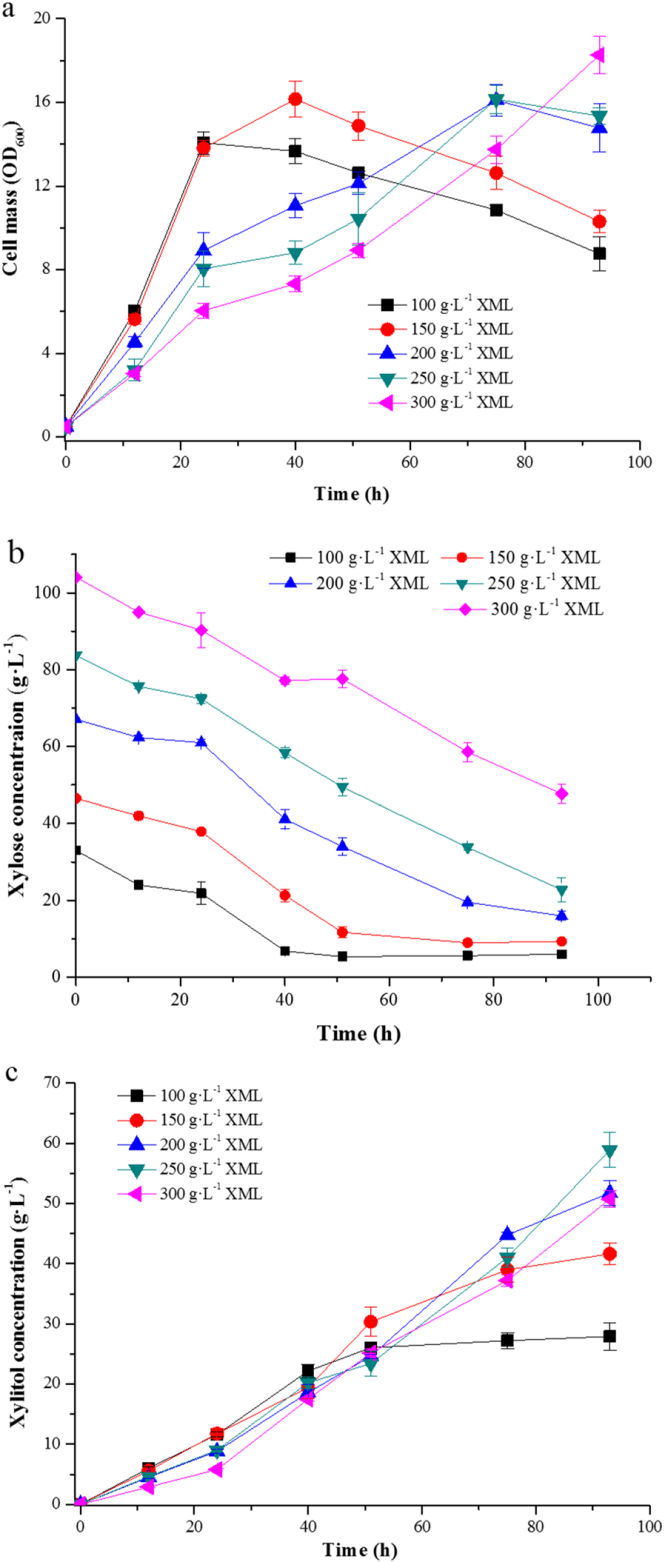
Effect of XML concentration on the fermentation process. **a** Effect on cell growth. **b** Effect on xylose concentration. **c** Effect on xylitol concentration. Error bars indicate standard deviations from the means of triplicate biological replicates

Having optimized pH and temperature, additional experiments were conducted to investigate the effects of XML concentration on cell growth, xylose consumption and xylitol production (Fig. 5). Cells grew rapidly and produced a relatively high xylitol yield at early times when the XML concentration was 100–150 g L^− 1^, but the cell mass began to decline after 24 h (Fig. 5a) due to glucose exhaustion (data not shown). After 96 h, the xylitol titer was significantly lower than that obtained using higher XML concentrations. Higher XML concentrations (200–300 g L^− 1^) produced higher xylitol titers, but the higher XML concentrations also hampered cell growth, probably because of the increased concentrations of inhibitors in the fermentation medium. Despite the slower growth rates, the final cell masses were similar to those obtained using lower XML concentrations, suggesting that strain XZX-B4ZG could adapt to this stress during fermentation. After 96 h, XZX-B4ZG cultured in fermentation medium containing XML 250 g L^− 1^ produced 58.9 g L^− 1^ xylitol with a yield of 98 %. Therefore, 250 g L^− 1^ was used as the optimal XML concentration for xylitol bioconversion.

Yeast extract, peptone and corn steep liquor were investigated as alternate nitrogen sources. The results showed that the lack of nitrogen sources seriously inhibited cell growth, but changing the nitrogen sources had minimal impacts on cell growth, xylose consumption rate, and xylitol titer (see Additional file 1: Fig. S6). Corn steep liquor seemed the best choice because its use led to the highest xylitol titer (53.6 g L^− 1^) with a yield of 95.22 % and because of its low cost.

### Enhanced production of xylitol from XML in a 5-L bioreactor

**Fig. 6:**
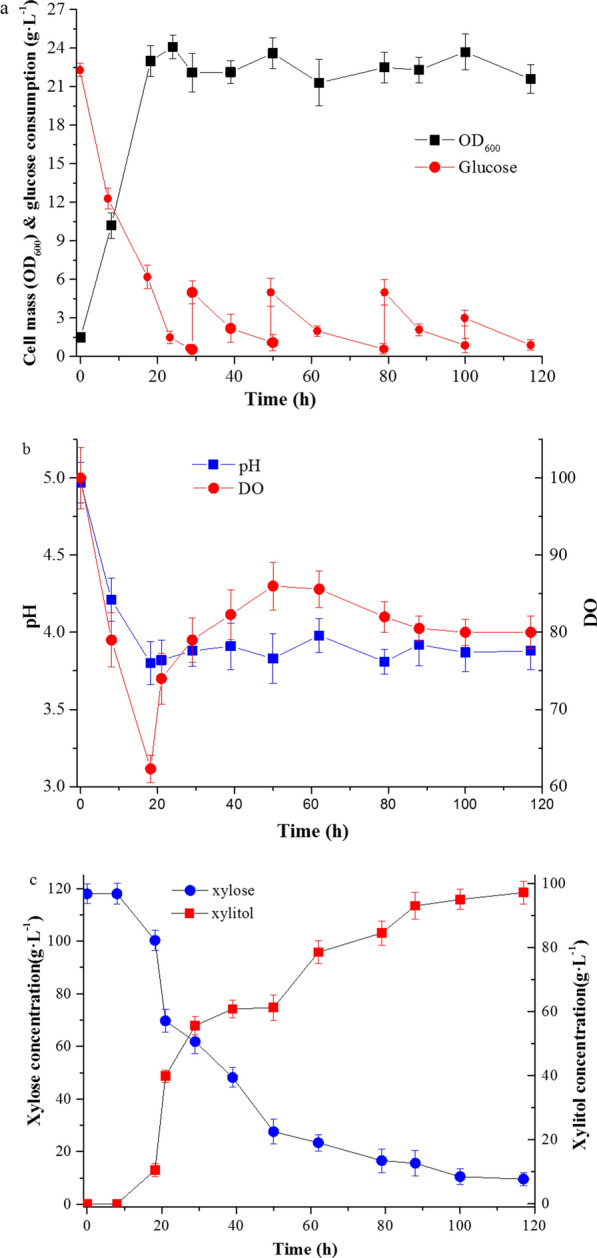
Xylitol production from XML in a 5-L fermenter. Representative result obtained at 35 ℃ with stirring at 700 r min^− 1^ and a aeration rate 4 v v^− 1^·min. **a** Cell mass growth and glucose consumption profiles, glucose was fed when concentration was below 1 g/L. **b** pH and dissolved oxygen (DO) profiles. **c** xylitol accumulation and xylose consumption profiles. Error bars indicate standard deviations from the means of triplicate biological replicates

To better assess the potential of strain XZX-B4ZG to produce xylitol from XML on an industrial scale, fermentation was performed in a 5-L bioreactor. Using conditions obtained from the optimization experiments, the cell mass peaked at ∼16 h (Fig. 6a), which was much earlier than that observed during shake-flask cultivation. Glucose was depleted at a correspondingly fast rate (Fig. 6a). The rapid initial growth rate led to a significant decrease in dissolved oxygen (DO) and the pH displayed a similar variation (Fig. 6b). When glucose was depleted and xylitol had begun to accumulate, the DO began to increase slowly (Fig. 6b). To generate reducing force NADPH for xylose reduction, glucose was added as co-substrate when its concentration was lower than 1 g L^− 1^. The xylitol accumulation rate increased after 8 h, leading to a final xylitol concentration of 97.1 g L^− 1^ with a productivity of 0.82 g L^− 1 ^h^− 1^ (Fig. 6c). These results suggest that fermentation of *C. tropicalis* XZX-B4ZG in a medium containing XML constitutes an efficient and simple bioprocess for xylitol production from XML, a low-cost biomass resource.

## Discussion

The xylose mother liquor is an abundant biomass by-product and a potential environmental pollutant in China. Here, we sought to apply metabolic engineering to industrial yeast strain for improving XML value. Genetic manipulations combined cofactor regeneration and process optimization resulted in engineered *C. tropicalis* efficiently overproducing xylitol from XML.

To improve the productivity of xylitol, two different coenzyme regeneration systems were created in *C. tropicalis*. Simultaneous expression of *YlZWF* and *YlGND* (strain XZX-B4ZG) significantly increased intracellular NADPH availability (Table 2), which was approximately 1.36-fold higher than that of XZX-B3. In contrast, individual expression of *YlZWF* (XZX-B4Z), *YlGND* (XZX-B4G) or *YlMAE* (XZX-B4M) only slightly increased intracellular NADPH availability (Table 2). Clearly, the reactions catalyzed by *YlZWF* and *YlGND* both limit flux through the pentose phosphate pathway, so that overexpression of each alone cannot substantially increase intracellular NADPH availability. Interestingly, all of the yeast strains containing augmented NADPH regeneration systems accumulated high amounts of NADP+. Previous studies have demonstrated cytosolic NADPH/NADP + ratios ranging from 15 to 60. These ratios drive the biosynthesis of fatty acids and nucleic acids, as well as the defense against oxidative stress [27, 28]. Our results revealed that overexpressing *YlZWF*, *YlGND* and/or *YlMAE* slightly decreased the NADPH/NADP ^+^ ratio, even though the intracellular NADPH supply increased. Further research is needed because augmentation of the pentose phosphate pathway is expected to increase the NADPH/NADP^+^ ratio [29].

**Table 2:** NADPH/NADP ^+^ contents of strains described in this study

Strains	NADPt (pmol/well)	NADPH (pmol/well)	NADP^+^ (pmol/well)	NADPH/NADP^+^
XZX-B3	24.53	22.52	2.01	11.20
XZX-B4Z	34.52	29.82	4.72	6.32
XZX-B4G	30.87	28.14	2.73	10.31
XZX-B4ZG	38.50	30.62	7.88	3.86
XZX-B4M	32.37	28.63	3.74	7.70

NADPt, total of NADP^+^ and NADPH concentrations

As shown in Fig. 2d, e and C. *tropicalis* utilized the glucose preferentially over xylose (the so-called carbon catabolite repression) in the medium containing a mixture carbon sources. The similar phenomenon was reported in *Kluyveromyces marxianus* [30] and *S. cerevisiae* [31]. This increased the difficulty for fermentation control and further limited xylitol productivity. Because xylose can not be used for growing of the engineered strain *C. tropicalis* XZX-B4ZG. Once the glucose was exhausted, xylitol accumulation is stopped due to lack of coenzyme for xylose reduction. To tackle this problem, the endogenous *KmHXK1* gene (encoding hexokinase 1 which involved in the glucose repression) in *K. marxianus* was knocked out and the exogenous xylose-specific transporter gene was overexpressed [30]. The engineered *K. marxianus* strain could co-utilize the xylose and glucose in fermentation medium for xylitol production. Construction of chimeric *C. tropicalis*-recombinant *Bacillus subtilis* co-cultures enabled high efficiency production of xylitol and the by-products in XML was used up as well, by taking advantage of combination the arabinose and galactose metabolism in *Bacillus subtilis* with high-efficiency xylitol biosynthesis in *C. tropicalis* [1]. Although the one-pot biotransformation has some unique advantages, constructing a “perfect” strain by metabolic engineering for monoculture is always attractive. In a previous report, the bacteria arabinose metabolic pathway was introduced into *XYL2*-disrupted *C. tropicalis* [32]. The resulting recombinant strain converted xylose into xylitol, without arabitol formation. Perhaps this strategy for removing arabinose from fermentation medium could also be taken for transforming XML into xylitol in largely industrial-scale production.

## Conclusions

In summary, *C. tropicalis* was engineered systematically to address the bottlenecks that sequentially limit xylitol biosynthesis from xylose. Genetic manipulations combined cofactor regeneration and process optimization resulted in engineered *C. tropicalis* efficiently overproducing xylitol from XML. Under the optimized conditions, the engineered strain, *C. tropicalis* XZX-B4ZG, produced 97.10 g L^− 1^ xylitol with 92.40 % conversion rate from 300 g L^− 1^ xylose mother liquor in a 5-L fermenter. The stepwise engineering strategy, as well as process investigation, can be used to rationally design cell factories for the eco-friendly use of xylose mother liquor and the production of highly valuable chemicals.

## Materials and methods

### Strains, media and culture conditions

The strains and plasmids used in this work are listed in Table 1. *C. tropicalis* XZX, a uracil auxotrophic derivative of *C. tropicalis* ATCC 20,336, was used as the parent strain for genetic manipulation [19]. Minimal mediun (MM; 6.7 g L^− 1^ yeast nitrogen base, 20 g L^− 1^ glucose and 10 g L^− 1^ (NH_4_)_2_SO_4_) and supplemented medium (SM; MM supplemented with uracil at a concentration of 0.06 g L^− 1^) with or without 5-fluoroorotic acid (5-FOA) was used to screen transformants generated during the genetic manipulation of *C. tropicalis* XZX. Fermentation medium (5 g L^− 1^ yeast extract containing xylose and/or glucose at the indicated concentration) and XML medium (5 g L^− 1^ yeast extract containing XML at the indicated concentration) were used to evaluate the xylitol production performance of engineered strains. *E. coli* JM109 (TaKaRa, Dalian, China), which was used as the host for all genetic manipulations, was cultured at 37 °C in LB medium (5 g L^− 1^ yeast extract, 10 g L^− 1^ peptone, 10 g L^− 1^ NaCl) supplemented with 100 µg mL^− 1^ ampicillin. Solid media were prepared by adding agar to a final concentration of 20 g L^− 1^. The XML used in this study was supplied by Futian Pharmaceutical Co., Ltd. (Dezhou, Shandong Province, China).

### Genetic manipulations

Deletion of *XYL2*. The *XYL2* gene (GenBank accession number: DQ201637.1) was sequentially deleted using the method described in our previous work [19]. The procedure is outlined in Additional file 1: Fig. S7. Firstly, the *XYL2* gene was amplified from *C. tropicalis* XZX genomic DNA using PCR using primers UXYL2 and DXYL2 (Table 3), and ligated into the plasmid vector pMD 18-T Simple (TaKaRa, Dalian, China) to yield plasmid Ts-*XYL2*. Then, inverse PCR with primers rUXYL2 and rDXYL2 (Table 3) was used to eliminate the middle region of *XYL2* (approximately 700 bp). The resulting PCR product (*XYL2I*-Ts-*XYL2I*) was digested with *Pst*I and *Xba*I and ligated to *gda324*-*URA3* cassettes (containing a functional yeast *URA3* gene flanked by a 324 bp gene disruption auxiliary sequence (*gda324*)) yielding plasmid Ts*-XYL2I*-*gda-URA3*. The disruption cassette for the sequential removal of both *XYL2* alleles was isolated from plasmid Ts-*XYL2I*-*gda*-*URA3* using PCR with primers UXYL2 and DXYL2. An established lithium chloride method [19] was used to transform *C. tropicalis* XZX with this cassette. Correct transformants were identified on MM plates, and confirmed by PCR and DNA sequencing. After removal of the *URA3* marker, the resulting strain was used for disruption of the second *XYL2* allele. A similar method was used to disrupt the second *XYL2* allele, generating the mutant strain *C. tropicalis* XZX-B4 (*xly2/xyl2*).

**Table 3:** Primers used in this study

Primers	Sequence^a^ (5′ to 3′)	Restriction sites
UXYL2	TAAATAGAACCCACGAATCCCT	
DXYL2	TTTACTCGTACTATGCACTCC	
rUXYL2	AACTGCAGAGTAGTGAATATCGGAACCACA	*Pst*I
rDXYL2	GCTCTAGAAACTTCCCAATTTCCGACT	*Xba*I
U2XYL2	CTAAATCCGGCCACTACCAC	
D2XYL2	CCAGCGTTACCAATTTGCAC	
rU2XYL2	AACTGCAGTCACCGAACTTCAAATCAGC	*Pst*I
rD2XYL2	GCTCTAGATGCCGTTGCCAGAACCAT	*Xba*I
UPGAPDH	CGGGATCCAACGTGGTATGGTTGT	*Xho*I
DPGAPDH	GCTCTAGATGTTTAAATTCTTTAATTGAGGGAT	*Bam*HI
UTGAPDH	CGACGCGTCTATCCAACAAACT	*Xba*I
DTGAPDH	CCGCTCGAGTCTGGTTTAGAAGTAGG	*Mlu*I
UZWF	GCTCTAGAATGACTGGCACCTTACCC	*Xba*I
DZWF	CGACGCGTCACGAGGAGCCCTT	*Xba*I
UGND	GCTCTAGAATGACTGACACTTCAAAC	*Mlu*I
DGND	CGACGCGTTTAAGCATCGT	*Xba*I
UMAE	GCGTCGACACCCGATTTCAAAAGTGCAGA	*Mlu*I
DMAE	CGACGCGTCTAGTCGTAATCCC	*Xba*I
YlZWF-F	AAGAACACCATTTCCAAC	For RT-qPCR
YlZWF-R	GATGATTCCAATGTCGTT	For RT-qPCR
YlGND-F	CCAACACGAATACTAACA	For RT-qPCR
YlGND-R	TTAACGAGCAGAATGATT	For RT-qPCR
YlMAE-F	AACGAGGTGCTCTACTAC	For RT-qPCR
YlMAE-R	CTGAGTCGGTGTATAGATGAT	For RT-qPCR

^a^Restriction sites introduced into the primers are underlined

Construction of coenzyme regeneration systems in *C. tropicalis XZX-B4*. The promoter and terminator of the glyceraldehyde-3-phosphate dehydrogenase gene (*GAPDH*, GenBank accession number: HQ171163.1) were amplified from the *C. tropicalis* ATCC 20,336 genome using PCR with primers UPGAPDH and DPGAPDH, and UTGAPDH and DTGAPDH, respectively (Table 2). The terminator fragment was inserted into pMD 18-T (TaKaRa, Dalian, China) to generate plasmid Tm-*T*_*GAPDH*_. The promoter fragment was digested with *Xba*I and *Bam*HI, and inserted into similarly digested Tm-*T*_*GAPDH*_ to generate plasmid Tm-*P*_*GAPDH*_-*T*_*GAPDH*_. The open reading frame of the 6-phosphate-glucose dehydrogenase gene (*YlZWF*, GenBank accession number: CP017557.1) from *Yarrowia lipolytica* CICC 31,251 was amplified using PCR with primers UZWF and DZWF and then inserted into Tm-*P*_*GAPDH*_-*T*_*GAPDH*_ between its *Mlu*I and *Xba*I sites to generate plasmid Tm-*P*_*GAPDH*_-*YlZWF*-*T*_*GAPDH*_. This plasmid was digested with *Hin*dIII and *Bam*HI to generate the *P*_*GAPDH*_-*YlZWF*-*T*_*GAPDH*_ cassette. After blunting its sticky ends using *pfu* DNA polymerase (BBI, Shanghai, China), the cassette was inserted into plasmid Ts*-XYL2I*-*gda-URA3* that had been digested with *Xba*I and had its sticky ends blunted, yielding plasmid Ts*-XYL2I*-*gda-URA3-P*_*GAPDH*_-*YlZWF*-*T*_*GAPDH*_. A 6.1 kb *YlZWF* expression cassette (*XYL2I*-*gda-URA3-P*_*GAPDH*_-*YlZWF*-*T*_*GAPDH*_*-XYL2I*) was obtained using PCR with primers UXYL2 and DXYL2. This cassette was inserted into *C. tropicalis* XZX-B4 using the lithium chloride method (the resulting strain was designated as *C. tropicalis* XZX-B4Z), and then the marker gene was removed to produce the strain *C. tropicalis* XZX-B4Zt.

A similar method was used to construct Ts*-XYL2II*-*gda-URA3-P*_*GAPDH*_-*YlGND*-*T*_*GAPDH*_, a plasmid containing an expression cassette for the 6-phosphate-gluconic acid dehydrogenase gene (*YlGND*, GenBank accession number: CP017554.1) from *Y. lipolytica*. In short, the *YlGND* open reading frame was amplified from *Y. lipolytica* CICC 31,251 using PCR with primers UGND and DGND. The resulting PCR fragment was inserted between the *Mlu*I and *Xba*I sites of Tm-*P*_*GAPDH*_-*T*_*GAPDH*_ to obtain plasmid Tm-*P*_*GAPDH*_-*YlGND*-*T*_*GAPDH*_. This plasmid was digested with *Hin*dIII and *Eco*RI to obtain the *P*_*GAPDH*_-*YlGND*-*T*_*GAPDH*_ cassette, which was inserted into the *Xba*I site of plasmid Ts*-XYL2II*-*gda-URA3* using a blunt-end ligation technique. This plasmid was designated Ts*-XYL2II*-*gda-URA3-P*_*GAPDH*_-*YlGND*-*T*_*GAPDH*_. The *YlGND* expression cassette *XYL2II*-*gda-URA3-P*_*GAPDH*_-*YlGND*-*T*_*GAPDH*_*-XYL2II*, which was obtained using PCR with primers U2XYL2 and D2XYL2, was inserted into *C. tropicalis* XZX-B4 and XZX-B4Zt using the lithium chloride method, respectively. This process generated strain *C. tropicalis* XZX-B4G and XZX-B4ZG.

The *YlMAE* open reading frame (GenBank accession number: XM-504112.1), which encodes the malic enzyme, was amplified from *Y. lipolytica* CICC 31,251 genomic DNA using PCR with primers UMAE and DMAE. The *YlMAE* expression cassette *XYL2I*-*gda-URA3-P*_*GAPDH*_-*YlMAE*-*T*_*GAPDH*_*-XYL2I* was constructed using the method described above (see Additional file 1: Fig. S3a). Finally, the cassette was used to transform *C. tropicalis* XZX-B4, generating *C. tropicalis* XZX-B4M.

### Shake-flask experiments

To assess *XYL2* gene function and the NADPH regeneration systems, strains were pre-cultured for 48 h in 100-mL shake flasks containing 20 mL of SM medium. These cultures were used to inoculate SM (glucose or xylose as a sole carbon source). Cell density was determined using the absorbance of the culture at 600 nm (OD_600_), which was measured with a spectrophotometer. Concentrations of xylose and xylitol were determined using HPLC.

For xylitol production, strains were initially cultured for 48 h in YPD medium (10 g L^− 1^ yeast extract, 20 g L^− 1^ peptone, 20 g L^− 1^ glucose) at 30 °C and 200 rpm. These seed cultures were used to inoculate 250-mL flasks containing 100 mL of fermentation medium or XML medium to the indicated starting OD_600_ value. During fermentation, the concentrations of xylose and xylitol and the cell density were determined at appropriate intervals.

### Bioreactor experiments

A single colony of *C. tropicalis* XZX-B4ZG was used to inoculate 20 mL of YPD medium in a 100-mL shake flask. The resulting culture was incubated on a rotary shaker at 30 °C and 200 rpm. This seed culture was used to inoculate a 250-mL shake flask containing 50 mL of optimized seed medium (YPD supplemented with 200 g L^− 1^ XML). After incubation for 70 h at 30 °C and 200 rpm, about 20 % (v/v) of this seed culture were transferred into a 5-L bioreactor (Bailun Co., China) containing 2.5 L of fermentation medium supplemented with 5 g L^− 1^ corn steep liquor and 300 g L^− 1^ XML. The initial pH was 5.0, and the pH during the fermentation was not controlled. Fermentations for xylitol production were performed at 35 °C and 700 rpm, and air was supplied at 4 vvm. In each run, glucose was added at a final concentration of 5 g L^− 1^ at 29, 50 and 79 h, 3 g L^− 1^ at 100 h to the bioreactor (glucose concentration lower than 1 g L^− 1^). Samples were collected at appropriate intervals and the concentrations of residual sugars and xylitol were determined using HPLC.

### RNA and cofactor levels

Relative *YlZWF*, *YlGND* and *YlMAE* messenger RNA (mRNA) levels in *C. tropicalis* were measured using real-time quantitative PCR (RT-qPCR) as previously described [19]. The intracellular of NADPH and NADP^+^ concentrations were measured using the NADP/NADPH Quantification Colorimetric Kit (Bio Vision, Bay Area, USA) as recommended by the manufacturer. Yeast cells were grown in SM and harvested during the appropriate growth period.

### Analytical methods

To quantify the concentrations of furfural, d-glucose, l-arabinose, d-xylose and xylitol, diluted supernatants were analyzed using an Agilent 1260 HPLC system (Agilent Technologies Inc., Santa Clara, CA) equipped with refractive index detector at 35 °C and an Aminex HPX-87 H cation exchange column (300 mm × 7.8 mm). The column was eluted with 5 mM H_2_SO_4_ (flow rate 0.5 mL min^− 1^) at 50 °C temperature. Before use, the mobile phase was purified by vacuum filtration through a 0.22 μm microfiltration membrane and trapped air was removed using an ultrasonic cleaner. Prior to analysis, 1 mL of the fermentation broth or XML was centrifuged at 10,000*g* for 5 min, and the supernatant were filtered through a 0.22 μm pore diameter cellulose acetate membrane. The concentration of acetate, furfural and 5-hydroxymethyl furfural in XML was determined as previously reported [1, 18, 26].

### Statistical analysis

Data are presented as means ± SD. The statistically significance of differences was analyzed using Student’s *t* test. Differences resulting in values of *P* < 0.05 were considered significant.

## Supplementary Information

**Additional file 1: Figure S1.** Growth curve of *C. tropicalis* XZX in XML medium. A colony of XZX was pre-culture in YPD medium, and diluted to a density of 0.5 at 600 nm (OD_600_) in 250 mL shake flasks with 50 mL XML medium (5 g·L^-1^ yeast extract, 200 g·L^-1^ XML) at 200 rpm and 30 °C. **Figure S2.** Confirmation of growth phenotype using xyloseas the sole carbon source. XZX, *C.tropicalis* XZX (parent strain); XZX-B2, XZX with one *XYL2* allele deleted; XZX-B4, XZX with both *XYL2* alleles deleted. **Figure S3.** Verification of *C.* *tropicalis *mutants using PCR. (a) Structure of integration cassette used to overexpress *YlMAE*. Similar cassettes were used to overexpress *YlZWF* and *YlGND*. (b) Verification of XZX-B4 and XZX-B4Zt by PCR using primers UXYL2 and UXYL2.M1: DL15 000 DNA marker; M2: DL5 000 DNA marker; lane 1: XZX-B4; lane 2: XZX-B4; (c) Verification of XZX-B4ZG by PCR. M1: DL15 000 DNA marker; M2: DL5 000 DNA marker; lane 1: XZX B4Z; lane 2: XZX B4; (d) Verificationof XZX-B4M by PCR. M1: DL15 000 DNA marker; M2: DL5 000 DNA marker; lane 1: XZX B4; lane 2: XZX-B4M. **Figure S4.** Evaluation of *YlZWF*, *YlGND* and *YlMAE* expression using qPCR. Evaluation of: (a) *YlZWF *expression by two transformants of *C. tropicalis* XZX-B4Z, compared with control strain XZX; (b) *YlGND* expression by two transformants of *C. tropicalis *XZX-B4ZG, compared with control strain XZX; and (c) *YlMAE *expression by two transformants of *C. tropicalis* XZX-B4M,compared with control strain XZX; Error bars indicate standard deviations from the means of triplicate biological replicates. **Figure S5.** Growth curve of XZX-B3, XZX-B4Z and XZX-B4ZG in fermentation medium (6.7 g·L^-1^ yeast nitrogen base, 10 g·L^-1^glucose, 50 g·L^-1^ xylose, 10 g·L^-1^ (NH_4_)_2_SO_4_, 0.06 g·L^-1 ^uracil and 2 g·L^-1^ furfural). **Figure S6.** Effect of nitrogen sources on xylitol production by* C. tropicalis* XZX-B4ZG in shake flasks. The experiment was done in 250 mL shake flasks with 100 mL fermentation medium (250 g·L^-1^ XML and 5 g·L^-1^ nitrogen sources, pH 5.0) at 200 rpm and 35°C. (a): Effect on cell growth (b): Effect on xylose concentration (c) Effect on xylitol concentration. **Figure S7.** *XYL2 *deletion in *C.* *tropicalis* XZX. Schematic depiction of the sequential disruption of the two* XYL2* alleles in *C. tropicalis* using miniature disruption cassettes. **Table S1.** Performance of fermentations using 100 g·L^-1^ XML as substrate.

## Data Availability

All data generated or analyzed during this study are included in this published article and its supplementary information files.
