# Supplementary material for: Stepwise metabolic engineering of Candida tropicalis for efficient xylitol production from xylose mother liquor
Source: Microb Cell Fact. 2021 May 25;20:105. doi: 10.1186/s12934-021-01596-1 (PMC8147403; doi:10.1186/s12934-021-01596-1)
Supplement: Supplementary file 1 — Additional file 1: Figure S1. Growth curve of C. tropicalis XZX in XML medium. A colony of XZX was pre-culture in YPD medium, and diluted to a density of 0.5 at 600 nm (OD600) in 250 mL shake flasks with 50 mL XML medium (5 g·L-1 yeast extract, 200 g·L-1 XML) at 200 rpm and 30 °C. Figure S2. Confirmation of growth phenotype using xyloseas the sole carbon source. XZX, C.tropicalis XZX (parent strain); XZX-B2, XZX with one XYL2 allele deleted; XZX-B4, XZX with both XYL2 alleles deleted. Figure S3. Verification of C. tropicalis mutants using PCR. (a) Structure of integration cassette used to overexpress YlMAE. Similar cassettes were used to overexpress YlZWF and YlGND. (b) Verification of XZX-B4 and XZX-B4Zt by PCR using primers UXYL2 and UXYL2.M1: DL15 000 DNA marker; M2: DL5 000 DNA marker; lane 1: XZX-B4; lane 2: XZX-B4; (c) Verification of XZX-B4ZG by PCR. M1: DL15 000 DNA marker; M2: DL5 000 DNA marker; lane 1: XZX B4Z; lane 2: XZX B4; (d) Verificationof XZX-B4M by PCR. M1: DL15 000 DNA marker; M2: DL5 000 DNA marker; lane 1: XZX B4; lane 2: XZX-B4M. Figure S4. Evaluation of YlZWF, YlGND and YlMAE expression using qPCR. Evaluation of: (a) YlZWF expression by two transformants of C. tropicalis XZX-B4Z, compared with control strain XZX; (b) YlGND expression by two transformants of C. tropicalis XZX-B4ZG, compared with control strain XZX; and (c) YlMAE expression by two transformants of C. tropicalis XZX-B4M,compared with control strain XZX; Error bars indicate standard deviations from the means of triplicate biological replicates. Figure S5. Growth curve of XZX-B3, XZX-B4Z and XZX-B4ZG in fermentation medium (6.7 g·L-1 yeast nitrogen base, 10 g·L-1glucose, 50 g·L-1 xylose, 10 g·L-1 (NH4)2SO4, 0.06 g·L-1 uracil and 2 g·L-1 furfural). Figure S6. Effect of nitrogen sources on xylitol production by C. tropicalis XZX-B4ZG in shake flasks. The experiment was done in 250 mL shake flasks with 100 mL fermentation medium (250 g·L-1 XML and 5 g·L-1 nitrogen sources, pH 5.0) at [file 12934_2021_1596_MOESM1_ESM.docx]

**Stepwise metabolic engineering of *Candida tropicalis* for efficient xylitol production from xylose mother liquor**

Lihua Zhang^1^, Zhen Chen^1^, Junhua Wang^1^, Wei Shen^1^, Qi Li^1*^, Xianzhong Chen^1*^

^1^Key Laboratory of Industrial Biotechnology, Ministry of Education, & School of Biotechnology, Jiangnan University, Wuxi 214122, People’s Republic of China

*Corresponding author:

Qi Li, *E-mail* address: liqi@jiangnan.edu.cn, Tel: +86-0510-85918176

Xianzhong Chen, *E-mail* address: xzchen@ jiangnan.edu.cn, Tel: +86-510-85918122, Fax: +86-510-85918122

**
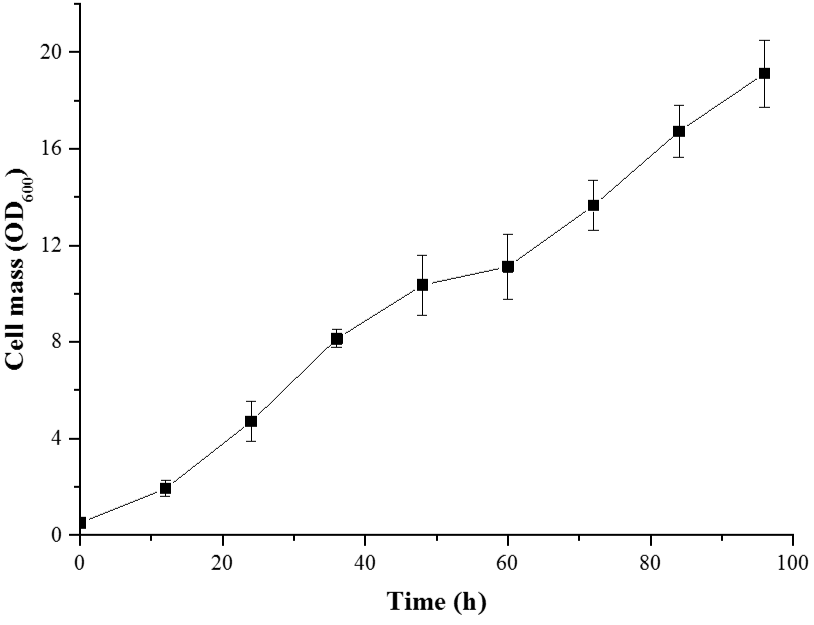
**

**Fig. S1** Growth curve of *C. tropicalis* XZX in XML medium. A colony of XZX was pre-culture in YPD medium, and diluted to a density of 0.5 at 600 nm (OD_600_) in 250 mL shake flasks with 50 mL XML medium (5 g·L^-1^ yeast extract, 200 g·L^-1^ XML) at 200 rpm and 30°C


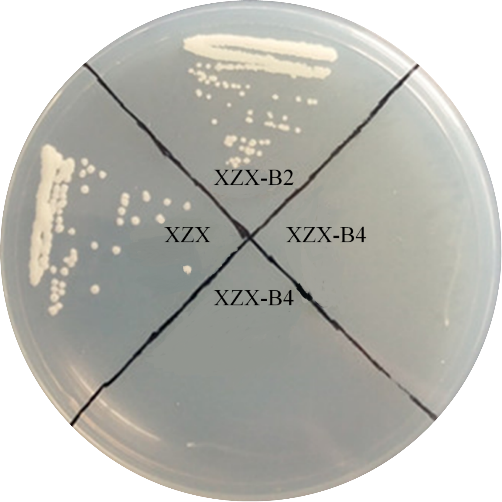


**Fig. S2** Confirmation of growth phenotype using xylose as the sole carbon source. XZX, *C. tropicalis* XZX (parent strain); XZX-B2, XZX with one *XYL2* allele deleted; XZX-B4, XZX with both *XYL2* alleles deleted


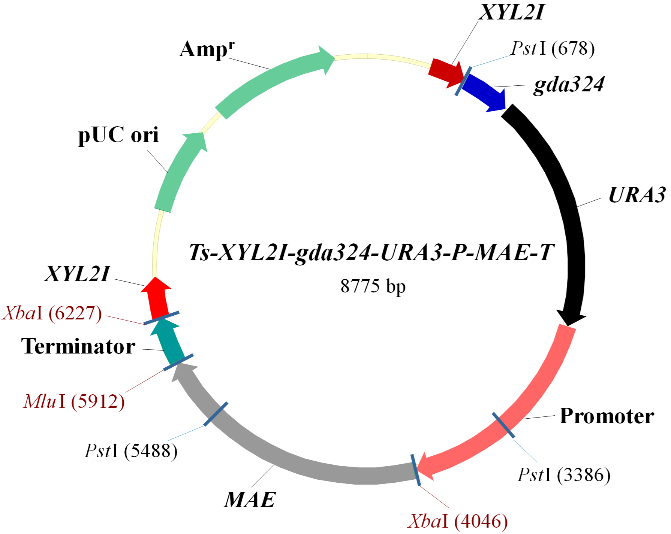


|  | a |  |
| --- | --- | --- |


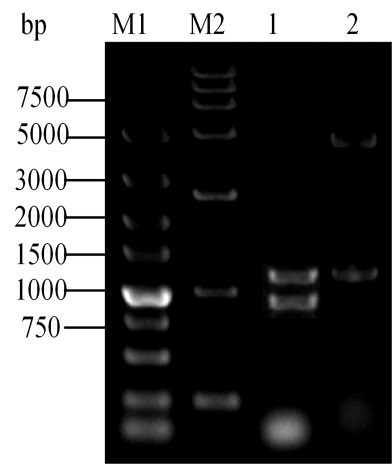

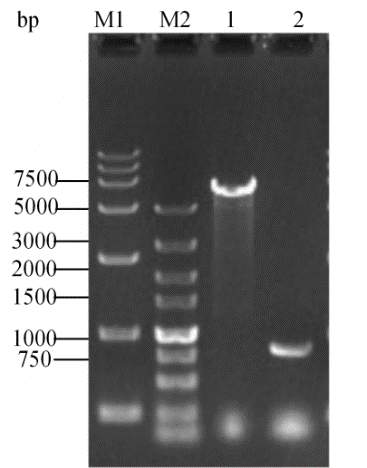

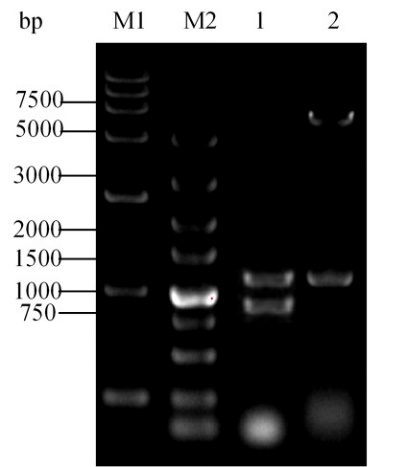


| b | c | d |
| --- | --- | --- |

**Fig. S3** Verification of *C.* *tropicalis* mutants using PCR. (a) Structure of integration cassette used to overexpress *YlMAE*. Similar cassettes were used to overexpress *YlZWF* and *YlGND*. (b) Verification of XZX-B4 and XZX-B4Zt by PCR using primers UXYL2 and UXYL2. M1: DL15 000 DNA marker；M2: DL5 000 DNA marker；lane 1: XZX-B4；lane 2: XZX-B4; (c) Verification of XZX-B4ZG by PCR. M1: DL15 000 DNA marker；M2: DL5 000 DNA marker；lane 1: XZX B4Z; lane 2: XZX B4; (d) Verification of XZX-B4M by PCR. M1: DL15 000 DNA marker；M2: DL5 000 DNA marker；lane 1: XZX B4; lane 2: XZX-B4M

**





**

**Fig. S4** Evaluation of *YlZWF*, *YlGND* and *YlMAE* expression using qPCR. Evaluation of: (a) *YlZWF* expression by two transformants of *C. tropicalis* XZX-B4Z, compared with control strain XZX; (b) *YlGND* expression by two transformants of *C. tropicalis* XZX-B4ZG, compared with control strain XZX; and (c) *YlMAE* expression by two transformants of *C. tropicalis* XZX-B4M, compared with control strain XZX; Error bars indicate standard deviations from the means of triplicate biological replicates

**
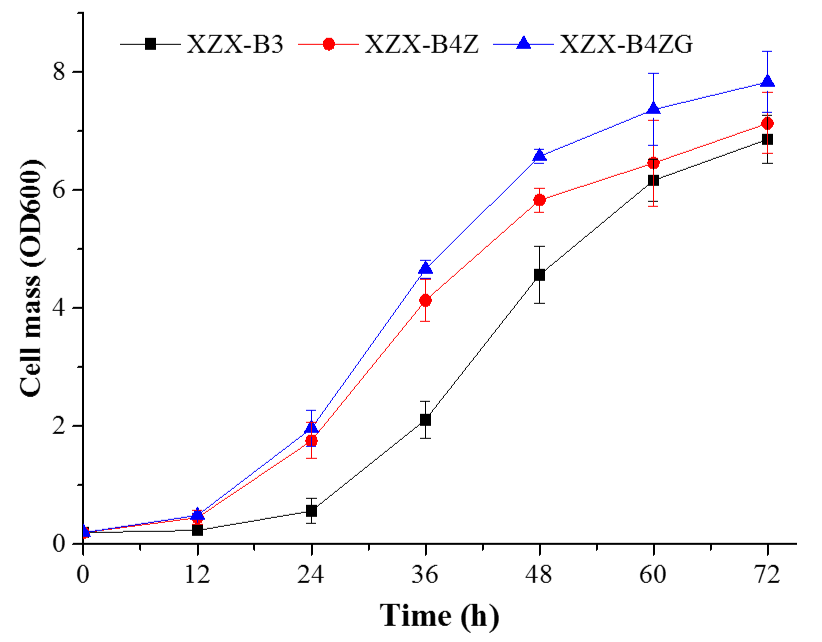
**

**Fig. S5** Growth curve of XZX-B3, XZX-B4Z and XZX-B4ZG in fermentation medium (6.7 g·L^-1^ yeast nitrogen base, 10 g·L^-1^ glucose, 50 g·L^-1^ xylose, 10 g·L^-1^ (NH_4_)_2_SO_4_, 0.06 g·L^-1^ uracil and 2 g·L^-1^ furfural)









**Fig. S6** Effect of nitrogen sources on xylitol production by *C. tropicalis* XZX-B4ZG in shake flasks. The experiment was done in 250 mL shake flasks with 100 mL fermentation medium (250 g·L^-1^ XML and 5 g·L^-1^ nitrogen sources, pH 5.0) at 200 rpm and 35°C. (a): Effect on cell growth (b): Effect on xylose concentration (c) Effect on xylitol concentration

**
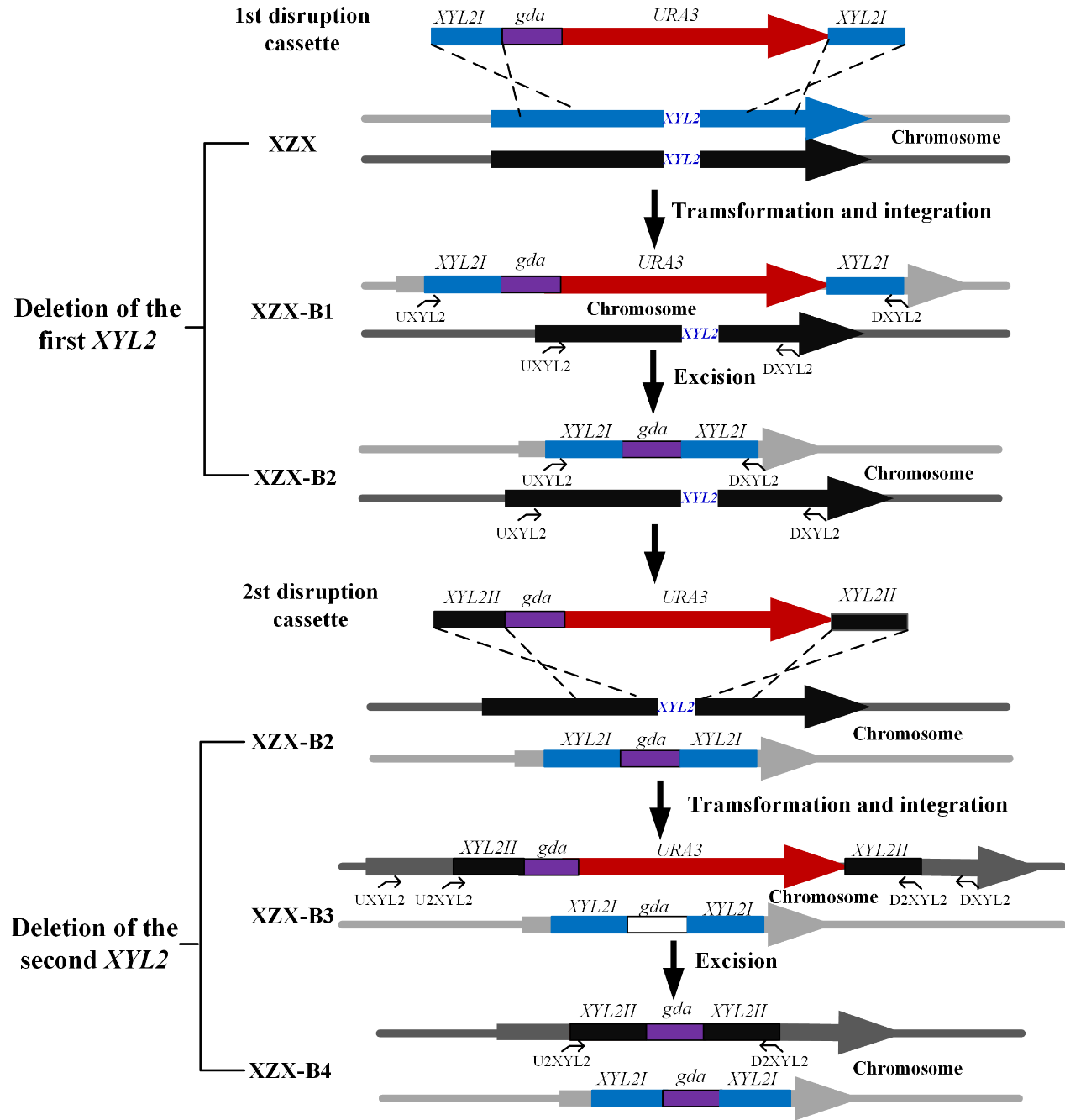
**

**Fig. S7** *XYL2* deletion in *C.* *tropicalis* XZX. Schematic depiction of the sequential disruption of the two *XYL2* alleles in *C. tropicalis* using miniature disruption cassettes

**Table S1** Performance of fermentations using 100 g·L^-1^ XML as substrate

| Strain | Xylose consumption (g·L^-1^) | Cell mass  (OD_600_) | Xylitol titer (g·L^-1^) | Yield  (% mol/mol) | *P* |
| --- | --- | --- | --- | --- | --- |
| XZX | 27.3±2.14 | 13.8±0.78 | 12.3±1.08 | 44.5 |  |
| XZX-B3 | 32.2±1.72 | 14.5±1.1 | 23.1±1.22 | 70.8 | <0.05 |
| XZX-B4ZG | 29.1±1.45 | 14.1±0.96 | 25.9±1.34 | 87.8 | <0.05 |

Values reported are the means (± standard deviation) of triplicate assays**.** Statistically significant difference was analyzed by Student’s *t* test. *P* < 0.05 was considered significant.
